# Initial Steps towards a Multilevel Functional Principal Components Analysis Model of Dynamical Shape Changes

**DOI:** 10.3390/jimaging9040086

**Published:** 2023-04-18

**Authors:** Damian J. J. Farnell, Peter Claes

**Affiliations:** 1 School of Dentistry, Cardiff University, Cardiff CF14 4XZ, UK; 2 Medical Imaging Research Center, UZ Leuven, 3000 Leuven, Belgium; 3 Department of Electrical Engineering, Processing of Speech and Images (ESAT-PSI), KU Leuven, 3000 Leuven, Belgium; 4 Department of Human Genetics, KU Leuven, 3000 Leuven, Belgium

**Keywords:** multilevel principal components analysis (mPCA), dynamical shape changes

## Abstract

In this article, multilevel principal components analysis (mPCA) is used to treat dynamical changes in shape. Results of standard (single-level) PCA are also presented here as a comparison. Monte Carlo (MC) simulation is used to create univariate data (i.e., a single “outcome” variable) that contain two distinct classes of trajectory with time. MC simulation is also used to create multivariate data of sixteen 2D points that (broadly) represent an eye; these data also have two distinct classes of trajectory (an eye blinking and an eye widening in surprise). This is followed by an application of mPCA and single-level PCA to “real” data consisting of twelve 3D landmarks outlining the mouth that are tracked over all phases of a smile. By consideration of eigenvalues, results for the MC datasets find correctly that variation due to differences in groups between the two classes of trajectories are larger than variation within each group. In both cases, differences in standardized component scores between the two groups are observed as expected. Modes of variation are shown to model the univariate MC data correctly, and good model fits are found for both the “blinking” and “surprised” trajectories for the MC “eye” data. Results for the “smile” data show that the smile trajectory is modelled correctly; that is, the corners of the mouth are drawn backwards and wider during a smile. Furthermore, the first mode of variation at level 1 of the mPCA model shows only subtle and minor changes in mouth shape due to sex; whereas the first mode of variation at level 2 of the mPCA model governs whether the mouth is upturned or downturned. These results are all an excellent test of mPCA, showing that mPCA presents a viable method of modeling dynamical changes in shape.

## 1. Introduction

**Figure 1:**
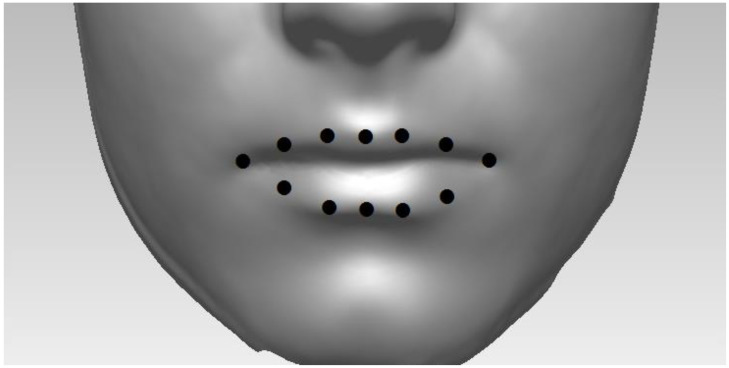
Illustration of lip shape, which is represented here by 12 landmark points placed on the 3D facial scans here.

Multivariate data occur when we have more than one “outcome” in our dataset. Biological shapes can be viewed as a form of multivariate data because shapes are conventionally represented by an abundance of geometric features or measurements. Traditionally, geometric measurements were taken directly onto subjects (e.g., using the Farkas System of Craniofacial Anthropometry [1]). However, today, most if not all biological shape analysis starts from indirect 2D or 3D image acquisitions. From these images, shapes can be represented by (e.g.,) manual placement of key landmark points (see Figure 1) or by semi-landmark methods, which also position landmark points regularly on an (often parametric) topological surface [2,3]. One may use methods such as principal components analysis (PCA) [2] to analyze such data. Between-group (bgPCA) [4,5] is an extension of standard PCA that carries out separate PCAs on (between-group) covariance matrices based on “group means” and (within group) covariance matrices based on individual shapes around these means. Multilevel PCA (mPCA) has been used by us [6,7,8,9,10,11,12,13] to analyze 3D facial shapes obtained from 3D facial scans; note that two-level multilevel PCA (mPCA) is equivalent to bgPCA. mPCA has been used previously to investigate changes by ethnicity and sex [6,7], facial shape changes in adolescents due to age [8,9], and the effects of maternal smoking and alcohol consumption on the facial shape of English adolescents [10]. Previous work also employed mPCA to treat time-related changes in facial shape during the act of smiling [11,12].

However, standard mPCA does not recognize that time is a continuous variable. A functional PCA (FPCA) approach is therefore more appropriate for such dynamical data [14,15,16]. FPCA is very similar to PCA, albeit with a preceding step where time-series are represented via some basis expansion (e.g., B-splines, wavelets, or Fourier series) and smoothing occurs [14]. However, the standard FPCA approach (such as single-level PCA) does not recognize clusters or multilevel structure within the subject population. Relatively few articles [17,18] have considered multilevel forms of FPCA (i.e., mFPCA) and certainly none have considered this in the context of facial shape.

Methods of analysis of sequences of 2D images include object tracking using point features [19], optical flow vectors [20,21], and convolutional neural networks [22]. By contrast, there are far fewer methods that have been applied to analyze time-dependent or dynamic changes in 3D shapes [23]. However, we note that dynamics changes can (e.g.,) play a strong role in the expression of disease in medicine, e.g., in 3D facial shapes [23] due to paralysis, stroke, cleft-lip and palate, or even schizophrenia. It is therefore imperative that we develop such methods in order to maximize the potential of this important source of (dynamic) data.

Here, we wish to apply a form of mPCA that is inspired by such functional approaches (and shares some advantages with them) to study shape dynamics, where results of standard PCA provide a comparison. Monte Carlo simulated datasets are used to test these methods in the first instance, although we also apply them to the “smile” data of Refs. [11,12], captured using a 3D dynamical scanner. We describe both datasets and methods in more detail in the methods section. We then discuss our results before going on to consider the implications of this work.

## 2. Materials and Methods

### 2.1. Sine Wave Dataset

Two Monte Carlo simulated datasets are used here. Firstly, a very simple case is presented with just one outcome (such as a signal or simple time series) with two groups. Trajectories for each subject follow a sine wave. We will refer to this as the “Sine Wave” dataset. The period of the first group is set to 2 (with respect to arbitrary units of time) and has an amplitude of 0.5 (with respect to arbitrary units of distance), whereas the period of the second group is set to 1.5 and has an amplitude of 0.4. Small normally distributed random errors are added to the amplitude (magnitude = 0.03) for each subject in order to provide variation between “within-subject” variation for the two groups. The magnitude of within-subject variation is therefore approximately two orders of magnitude smaller than that of between-groups variation (as variance scales with the amplitude squared).

### 2.2. Blink Dataset

The second Monte Carlo dataset has 16 points in 2D (thus, 2 × 16 = 32 components) that delineate the boundary (roughly) of an eye during: (group 1) an entire blink; (group 2) an eye opening slightly, as if in surprise. We shall refer to this as the “Blink” dataset. Again, small normally distributed random errors are added to the amplitude (magnitude = 0.03) for each subject in order to provide variation between “within-subject” variation for the two groups. A two-level mPCA model (presented below) is used to analyze this data. In both cases, between-groups variation has a much larger magnitude than within-groups variation.

### 2.3. Smile Dataset

This dataset consisted of 3D video shape data during all phases of a smile, where 12 points are placed (and tracked) along the outer boundary of mouth, as shown in Figure 1. A 3DMD scanner was used to capture this 3D surface dynamical data. Sixty adult staff and students at Cardiff University, consisting of thirty one males and twenty nine females, were recruited for this study. There were between approximately 100 and 250 frames in total for each subject. All 3D shapes were centered to have a common origin. As described in Refs. [11,12], different phases of the smile were found for each subject separately by considering the normalized smile amplitudes. Including rest phases, these seven phases were found to be [11,12] rest pre-smile, onset acceleration, onset deceleration, apex, offset acceleration, offset deceleration, and rest post-smile. Ethical approval for this project was granted by the School of Dentistry Ethics Committee at Cardiff University, UK.

### 2.4. Functional Principal Components Analysis (FPCA)

The covariance function for (single-level) FPCA with respect to time variables s and t (time is a continuous variable) is presented generally by

(1) $$K\left(s,t\right)=E\left[\left\{X\left(s\right)-\eta \left(s\right)\right\}\left\{X\left(t\right)-\eta \left(t\right)\right\}\right],$$

where $\eta \left(t\right)$ is the mean shape function (e.g., in practice with respect to all subjects in the dataset). Note that we may write $K\left(s,t\right)$ as

(2) $$K\left(s,t\right)=\sum_{l}^{\infty }\theta_{l}\psi_{l}\left(s\right)\psi_{l}\left(t\right),$$

where $\theta_{l}$ are non-negative eigenvalues and $\psi_{l}\left(t\right)$ are the associated eigenfunctions. Finally, the expansion of any (new) dynamic shape function $X\left(t\right)$ is approximated by

(3) $$X\left(t\right)=\eta \left(t\right)+\sum_{l}^{m}a_{l}\psi_{l}\left(t\right),$$

where $a_{l}$ are scalar coefficients and m is often set to be finite. Component scores are standardized readily by finding, $a_{l}/\sqrt{\theta_{l}}$.

Here, we carry out an mPCA approach that is inspired by functional methods in that dynamical shape changes are approximated by spline fits. This step has some advantages: random errors should be reduced by smoothing; data might be sampled at regular specific time points by interpolation; and issues of missing data frames or irregular image capture are addressed. As explained in Appendix A, spline fits are carried of X. with respect to time for each subject separately at T regular time intervals for each subject. Examples of specific fits are also given in Appendix A. When the outcome is a scalar variable sampled at T regular time intervals (e.g., a signal or single point in 1D), we denote the outcome variable for each subject i as a vector $X^{i}$ (of size T), where each component of this vector for subject i is denoted $X_{t}^{i}$, and where the index t denotes each of the regularly sampled time points. The total number of subjects (i.e., the sample size) is given by n. The covariance matrix is therefore of size T×T and is given by

(4) $$K_{t_{1},t_{2}}=\frac{1}{n-1}\sum_{i}^{n}\left(X_{t_{1}}^{i}-\eta_{t_{1}}\right)\left(X_{t_{2}}^{i}-\eta_{t_{2}}\right),$$

where each component of this matrix is denoted $K_{t_{1},t_{2}}$, and η is the mean trajectory vector of dimension T ($\eta_{t_{1}}$ and $\eta_{t_{2}}$ are specific elements of η), where

(5) $$\eta =\frac{1}{n}\sum_{i}^{n}X^{i}.$$

Eigenvalues of Equation (4) are denoted $\theta_{l}$ and eigenvectors are denoted $\psi_{l}$. The expansion of any (new) trajectory vector $X^{new}$ is given by

(6) $$X^{new}=\eta +\sum_{l}^{m}a_{l}\psi_{l},$$

where, again, $a_{l}$ are scalar coefficients and m is often set to be a finite number. The components $a_{l}$ (referred to as “component scores” here) are found via: $a_{l}=\left(X^{new}-\eta \right)\cdot \psi_{l}$.

For multivariate shape data, shapes at each time point are themselves a vector rather than a scalar and this is explained in detail in Appendix A. Spline fits are carried out for each subject and for each 3D point component separately with respect to time. The size of the vector $X^{i}$ is given by Dim×M×T for each subject i. We denote the index of each element of this vector as $\left\{k,t\right\}$; components of this vector are therefore given by $X_{\left\{k,t\right\}}^{i}$. Elements of the covariance matrix are now written as

(7) $$K_{\left\{k_{1},t_{1}\right\},\left\{k_{2},t_{2}\right\}}=\frac{1}{n-1}\sum_{i}^{n}\left(X_{\left\{k_{1},t_{1}\right\}}^{i}-\eta_{\left\{k_{1},t_{1}\right\}}\right)\left(X_{\left\{k_{2},t_{2}\right\}}^{i}-\eta_{\left\{k_{2},t_{2}\right\}}\right).$$

η is again the mean vector. The diagonalization of this covariance matrix can become an intensive computational problem and so direct iteration or the Lanczos method can be used (as appropriate) to find the eigenvalues $\theta_{l}$ and the eigenvectors $\psi_{l}$. The expansion of any (new) trajectory vector $X^{new}$ may again be found via Equation (6).

This approach assumes that all components of all landmark points are correlated potentially with all other components and at all time points. Clearly, this is an inefficient approach, but it is simple and straightforward to implement. It is therefore used in these initial calculations.

### 2.5. Multilevel Functional Principal Components Analysis (mPCA)

Multilevel/hierarchical models allow clustering to be addressed by including between- and within-group variations at different levels of the model. For a simple two-level model (initially), we write our feature vector as $X^{i,j}$, where i indicates a specific instance or subject in group or cluster j (of p such groups). Again, we assume initially that the outcome is a scalar, and that the trajectory data have been sampled regularly at T time points. We write elements of the level 2 (within-group variation) covariance matrix $K^{2,j}$ for each group j as

(8) $$K_{t_{1},t_{2}}^{2,j}=\frac{1}{n_{j}-1}\sum_{i}^{n_{j}}\left(X_{t_{1}}^{i,j}-\eta_{t_{1}}^{j}\right)\left(X_{t_{2}}^{i,j}-\eta_{t_{2}}^{j}\right)\;.$$

$\eta^{j}$ is the mean for each group j, given by

(9) $$\eta^{j}=\frac{1}{n_{j}}\sum_{i}^{n_{j}}X^{i,j}.$$

However, a “common” covariance matrix is often assumed for mPCA (specifically here at level 2). For p such groups in total, we note that this is commonly written for mPCA as

(10) $$K^{2}=\frac{1}{p}\sum_{j}^{p}K^{2,j}.$$

An interesting point is that standard mPCA weights individual covariance matrices with a factor of 1/p in Equation (10) rather than a factor of $n_{j}/n$, which is the result for the maximum likelihood solution using an underlying multivariate normal distribution [9]. An advantage of this approach is that each group is treated equally, irrespective of its sample size. However, this will not fit the available data as well as the maximum likelihood solution and small sample size effects can occur (see “pathologies” below). The “grand mean” is now given by

(11) $$\eta =\frac{1}{p}\sum_{j}^{p}\eta^{j}.$$

$K^{2}$ can now be diagonalized, where $\theta_{l}^{2}$ are non-negative eigenvalues at level 2 and $\psi_{l}^{2}$ are the associated eigenvectors. By contrast, we now write elements of the covariance matrix at level 1 (between-group variation) as

(12) $$K_{t_{1},t_{2}}^{1}=\frac{1}{p-1}\sum_{j}^{p}\left(\eta_{t_{1}}^{j}-\eta_{t_{1}}\right)\left(\eta_{t_{2}}^{j}-\eta_{t_{2}}\right),$$

where $\theta_{l}^{1}$ are non-negative eigenvalues at level, and $\psi_{l}^{1}$ are the associated eigenvectors. Note that the number of non-zero eigenvalues will be limited to p−1 due to the finite number of groups p. The expansion of any (new) dynamic shape $X^{new}\;$ is given by

(13) $$X^{new}=\eta +\sum_{l}^{m_{1}}a_{l}^{1}\psi_{l}^{1}+\sum_{l}^{m_{2}}a_{l}^{2}\psi_{l}^{2}.$$

Again, $a_{l}^{1}$ and $a_{l}^{2}$ are scalar coefficients and $m_{1}$ and $m_{2}$ are set to be finite numbers. The coefficients $\left\{a_{l}^{1}\right\}$ and $\left\{a_{l}^{2}\right\}$ (again referred to as “component scores” here) are determined for mPCA by using a global optimization procedure in MATLAB. Component scores are again standardized readily by finding, $a_{l}^{1}/\sqrt{\theta_{l}^{1}}$ and $a_{l}^{2}/\sqrt{\theta_{l}^{2}}$.

This approach will work for cases where the outcome is a scalar (i.e., X is a vector of size T) or for multivariate dynamic shape data (i.e., X is a vector of size Dim×M×T). We write elements of the level 2 (within-group variation) covariance matrix $K^{2,j}$ for each group j as

(14) $$K_{\left\{k_{1},t_{1}\right\},\left\{k_{2},t_{2}\right\}}^{2,j}=\frac{1}{n_{j}-1}\sum_{i}^{n_{j}}\left(X_{\left\{k_{1},t_{1}\right\}}^{i,j}-\eta_{\left\{k_{1},t_{1}\right\}}^{j}\right)\left(X_{\left\{k_{2},t_{2}\right\}}^{i,j}-\eta_{\left\{k_{2},t_{2}\right\}}^{j}\right).$$

This is a matrix of size: Dim×M×T by Dim×M×T. The common level 2 covariance matrix is again given by Equation (10) and the “grand mean” by Equation (11). Elements of the covariance matrix at level 1 (between-group variation) are

(15) $$K_{\left\{k_{1},t_{1}\right\},\left\{k_{2},t_{2}\right\}}^{1}=\frac{1}{p-1}\sum_{j}^{p}\left(\eta_{\left\{k_{1},t_{1}\right\}}^{j}-\eta_{\left\{k_{1},t_{1}\right\}}\right)\left(\eta_{\left\{k_{2},t_{2}\right\}}^{j}-\eta_{\left\{k_{2},t_{2}\right\}}\right).\;.$$

The expansion of any (new) dynamic shape $X^{new}\;$ is again given by Equation (13). The extension to three or more levels is presented in Appendix B. We also note that if each subject is treated as a separate group, then this approach models the “nested” nature of these dynamical shape data in an efficient manner (i.e., analogous to a mixed model in which repeated measurements of shape over time are made for each subject). Indeed, it is likely that this approach is probably very similar to that presented in Ref. [17], albeit now also for multivariate data.

A problem with mPCA is that divisions between groups can occur purely due to random sampling (and so are wholly spurious) because such random differences over all variables become condensed at a given level of the multilevel model [4,13,24]. This can lead to apparently strong and erroneous divisions in component scores for mPCA at this level, which are even more pronounced than for PCA. A rough rule of thumb [13] is that the sample size per group in the training set must be larger than the overall size of the feature vector in order to avoid “pathologies” of mPCA-based methods. (The topic of such pathologies is discussed in more detail in the conclusion.) For the “Sine Wave” dataset, T=101 (thus the feature vector is of size 101 also) and so we set the sample size for all groups to be $n_{j}=1000$. Sample sizes per group in the test set are also set to be $n_{j}=1000$. For the “Blink” dataset, T=101; thus, the feature vector is of size 32×101=3201 and so we set the sample size for all groups to be $n_{j}=10,000$. Sample sizes per group in the test set are also set to be $n_{j}=10,000$. For the “Smile” dataset, we note that T=60 and so the feature vector is of size 60×12×3=2160. Unfortunately, data are limited in this case to 60 subjects in total only. However, this is adequate here as we wish to present a “proof-of-principle” calculation only for “real” data. Again, all calculations presented here were carried out in MATLAB R2021a.

## 3. Results

### 3.1. Sine Wave Dataset

**Figure 2:**
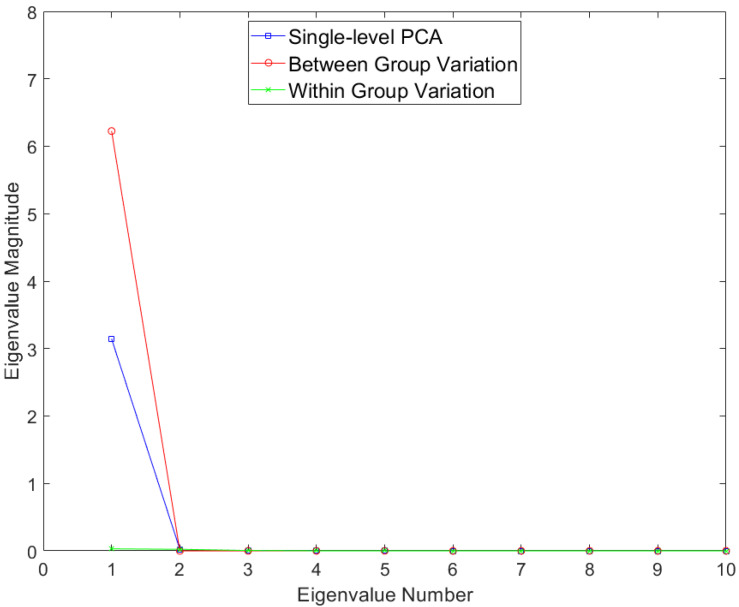
Eigenvalues for single-level PCA and mPCA for between-groups variation (level 1) and within-groups variation (level 2) for the Sine Wave dataset.

Results for the eigenvalues from mPCA and single-level PCA for the Sine Wave dataset are shown in Figure 2. There is only a single non-zero eigenvalue at level 1 (between-groups variation) via mPCA, which is what we expect as the number of groups in this simple simulation is two. By contrast, there are three non-zero eigenvalues at level 2 (within-groups variation) via mPCA, albeit with magnitudes less than approximately 0.03; this is to be expected as the between-group variation was set to be much larger than other (random) sources of variation. Three non-zero eigenvalues are also found for single-level PCA, which agrees well with results at level 2 (within-groups variation) via mPCA. The single large eigenvalue at level 1 via mPCA is also clearly echoed in the first eigenvalue for single-level PCA, as expected. Interestingly, the magnitude of this first eigenvalue is much lower for single-level PCA compared to level 1, mPCA; we speculate that this is because single-level PCA is not capturing all of the variation of the data. The other two eigenvalues for single-level PCA also have magnitude less than approximately 0.03.

**Figure 3:**
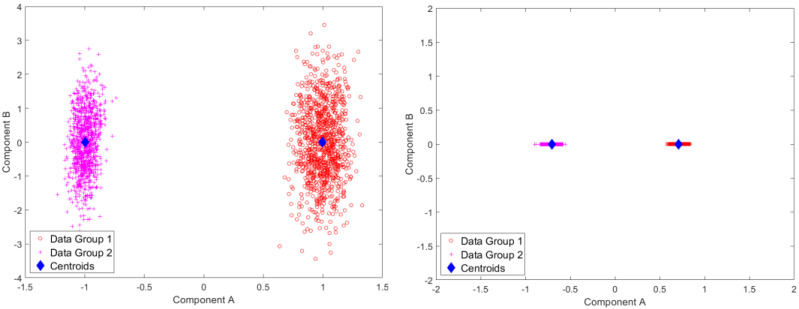
Results of standardized component scores for the test set of 1000 different trajectories per group in the test set for the Sine Wave dataset: (**left**) single-level, PCA; (**right**) level 1, mPCA. These results show strong clustering with respect to the two groups.

Results for standardized component scores via single-level PCA and mPCA are shown in Figure 3. We see that strong differentiation between groups is seen for mode 1 in single-level PCA. Strong differences between groups are also observed in Figure 3 at level 1 via mPCA. No strong difference in component scores between groups is seen at level 2 for mPCA, and centroids for these groups are congruent (not shown here).

**Figure 4:**
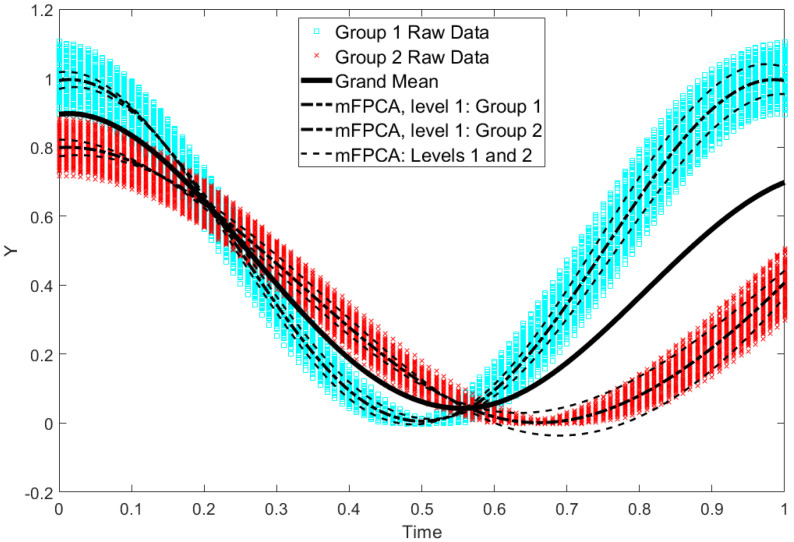
Data for the two groups in the test set of the Sine Wave dataset are shown by the green squares and red crosses (1000 trajectories per group). The overall “grand mean” is shown also. Model fits of mPCA (first mode at level 1 only denoted “mPCA, Level 1: Group 1/2”) based on the centroids in Figure 3 are shown also. Within-group (level 2) variation is added to the model fit for mPCA for each group (i.e., ±$1.96\times \sqrt{\theta_{1}^{2}\;}\times \psi_{1}^{2}$, denoted “mPCA: Levels 1 and 2”).

By using the centroids of standardized component scores shown in Figure 3, we may fit the single-level PCA model and mPCA at level 1 (between-group variation) only to the raw data in the test set. Results for mPCA are shown in Figure 4. Results for model fits via single-level PCA model and mPCA are found to capture mean trajectories with time for the two groups. These results for the two groups are also almost congruent at all time points for PCA and mPCA (not shown here). We also add an additional source of variation at level 2, namely, model fit for mPCA for each group plus/minus the first model at level 2, i.e., ±$1.96\times \sqrt{\theta_{1}^{2}\;}\times \psi_{1}^{2}$, which is essentially a 95% prediction interval with respect to this mode. We see that within-group variation around these two curves is being captured correctly (broadly). Presumably, even better correspondence would be obtained by adding in more modes at level 2. All in all, the Sine Wave dataset has been a successful test of the mPCA method.

### 3.2. Blink Dataset

**Figure 5:**
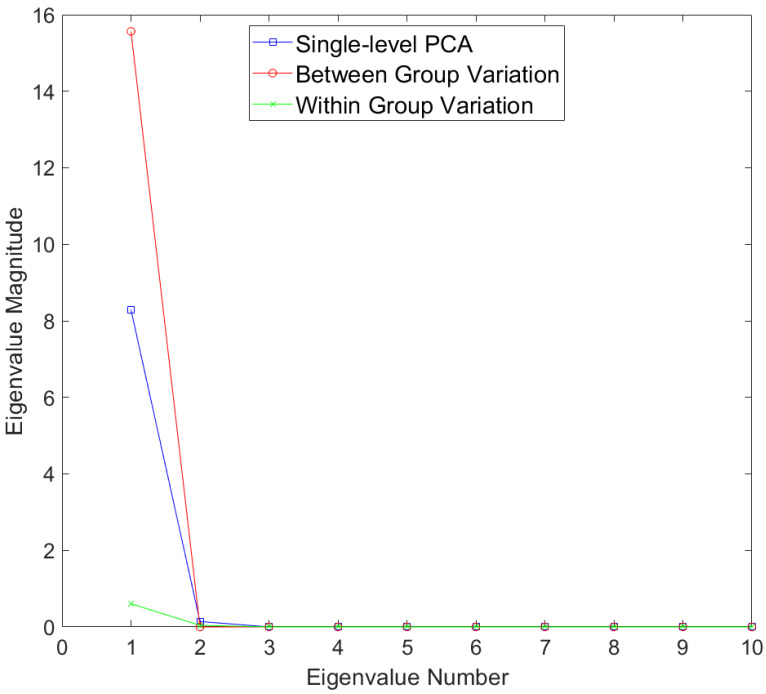
Eigenvalues for single-level PCA and mPCA for between-groups variation (level 1) and within-groups variation (level 2) for the Blink dataset.

Results for the eigenvalues from mPCA and single-level PCA for the Blink dataset are shown in Figure 5. Again, there is only a single non-zero eigenvalue at level 1 (between-groups variation) via mPCA, which, again, is what we expect as the number of groups in this simple simulation is just two (i.e., for “blinking” and “surprise” type trajectories). By contrast, there are two non-zero eigenvalues at level 2 (with-groups variation) via mPCA, although the magnitude of these eigenvalues is much smaller than the single eigenvalue at level 1, which again is as expected. Three non-zero eigenvalues are found also for single-level PCA, which also agrees well with results at level 2 (within-groups variation) via mPCA. The single large eigenvalue at level 1 via mPCA again is also clearly reflected in the first eigenvalue for single-level PCA. Interestingly again, the magnitude of this eigenvalue is much lower for single-level PCA.

**Figure 6:**
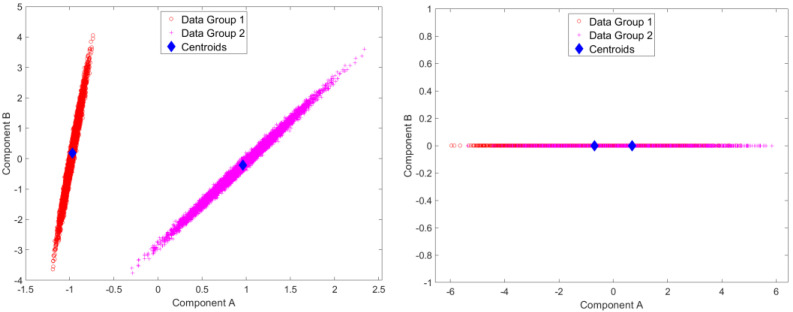
Results of standardized component scores for the test set of 10,000 different trajectories per group in the test set for the Blink dataset: (**left**) single-level PCA; (**right**) level 1, mPCA. These results again show strong clustering with respect to the two groups (i.e., “surprised” in group 1 and “blinking” in group 2.).

Results for standardized component scores via single-level PCA and mPCA are shown in Figure 6. We see that strong differentiation between groups is again seen just for mode 1 for single-level PCA. The magnitude of differences in centroids of standardized component scores for the two groups are of order two for single-level PCA and of order one at level 1 via mPCA, as shown in Figure 6. This suggests that strong differences occur between groups, although Figure 6 also clearly shows that there is much overlap between individual scores at level 1 via mPCA. We note that this level has a single non-zero eigenvalue only because the rank of the covariance matrix—and thus the maximum number of eigenvalues—is constrained to be no larger than the number of groups minus one (here, there are just two groups). Again, no strong difference in component scores between groups is seen at level 2 for mPCA, and centroids for these group are congruent (not shown here).

**Figure 7:**
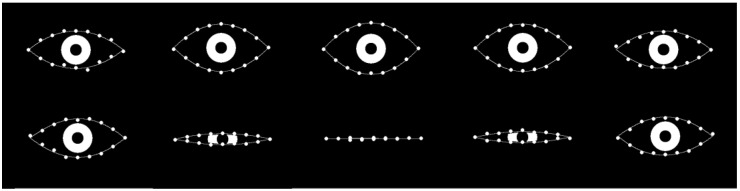
mPCA model fits (full lines) to entire trajectories (going from left to right in the images above) of the 16 2D landmark points of 2 specific examples chosen randomly from the test set: (upper row of images) from group 1: an eye “showing surprise,” i.e., the eye opens wider before returning to normal; (upper row of images) from group 2: an eye “blinking,” i.e., the eye closes before returning to normal. (The iris is added as an illustration only.)

Two specific cases from the test dataset (randomly chosen) may be explored via mPCA by using exactly the same two-level model for both cases. Here, we use just the single mode at level 1 only and no level 2 variation. The results are shown in Figure 7. We see that PCA correctly models the trajectories of all points delineating the boundary of the “eye” and at all stages of either the “surprised” expression or the “blinking” dynamic shape changes using the same model. Note that results of mPCA are shown by full lines in Figure 7, which are two separate cubic spline fits for the upper and lower boundaries of the eye with respect to the predicted 16 2D points from the mPCA model. Clearly, this might lead to some additional smoothing, although this is carried out to make it easier to visualize and interpret model results and to differentiate between them and the original data, shown by the points in Figure 7. (We believe that this is reasonable for these initial calculations and as a broad final check of model results.) Better model fits might be obtained by including additional modes from level 2, although we need to be careful not to overfit our model to the data. However, this is another excellent test of the viability of this method—in this case, also for multivariate data.

### 3.3. Smile Dataset

**Figure 8:**
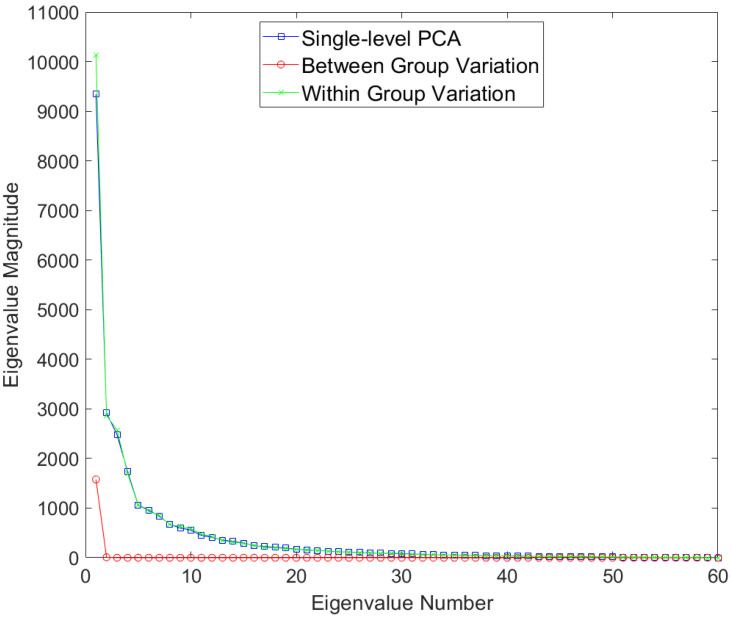
Eigenvalues for single-level PCA and mPCA for between-groups variation (level 1) and within-groups variation (level 2) for the Smile dataset.

Results for the eigenvalues from mPCA and single-level PCA for the Smile dataset are shown in Figure 8. Results of level 2 via mPCA are almost congruent with single-level PCA. The single eigenvalue at level 1 via mPCA is of much smaller magnitude than those eigenvalues at level 2. This is the first evidence that the magnitude of the difference in smile dynamics between males and females is small.

**Figure 9:**
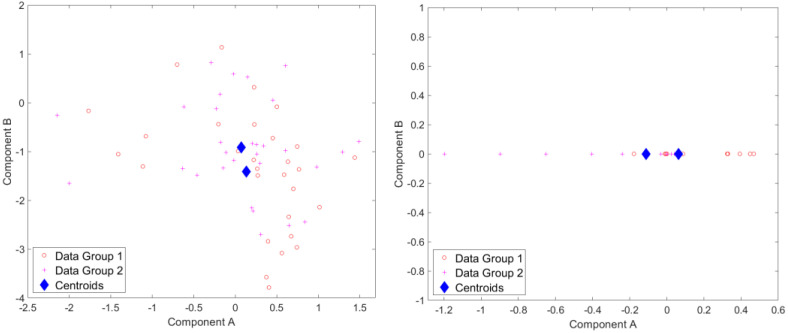
Results of standardized component scores for the Smile dataset: (**left**) single-level PCA; (**right**) level 1, mPCA. These results show only minor differences in dynamic 3D trajectories between males and females as the centroids for the two groups are quite close together.

Results for standardized component scores via single-level PCA and mPCA for the Smile dataset are shown in Figure 9. By contrast to the earlier simulated dataset, differences between groups (males and females) appear quite small for both single-level PCA and mPCA at level 1. This is yet more evidence that no strong differences in the 3D dynamics of smiles occur between men and women. Again, no strong difference in component scores between groups is seen at level 2 for mPCA and centroids for males and females are congruent (not showed here).

**Figure 10:**
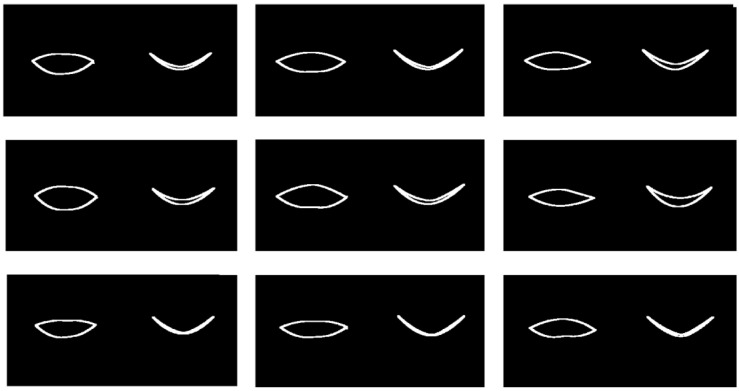
Visualization of the first mode of variation via mPCA at level 1 in the frontal/coronal plane (left) and horizontal plane (right) in each image. (**Left Column**) start of the smile; (**Middle Column**) mid-point (apex or plateau) of the smile; (**Right Column**) end of the smile. (**Top row**) mean shape trajectory; (**Middle Row**) mean shape trajectory minus $\sqrt{\theta_{1}^{1}}$ × first mode of variation via mPCA at level 1; (**Bottom Row**) mean shape trajectory plus $\sqrt{\theta_{1}^{2}}$ × first mode of variation via mPCA at level 1.

**Figure 11:**
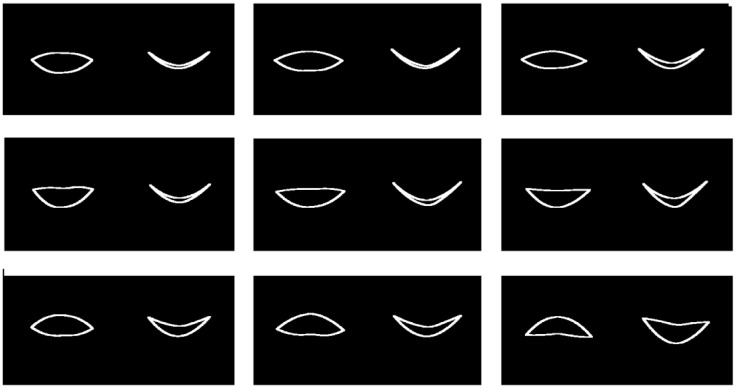
Visualization of the first mode of variation via mPCA at level 2 in the frontal/coronal plane (left) and horizontal plane (right) in each image. (**Left Column**) start of the smile; (**Middle Column**) mid-point (apex or plateau) of the smile; (**Right Column**) end of the smile. (**Top row**) mean shape trajectory; (**Middle Row**) mean shape trajectory minus $\sqrt{\theta_{1}^{2}}$ × first mode of variation via mPCA at level 2; (**Bottom Row**) mean shape trajectory plus $\sqrt{\theta_{1}^{2}}$ × first mode of variation via mPCA at level 2.

**Figure 12:**
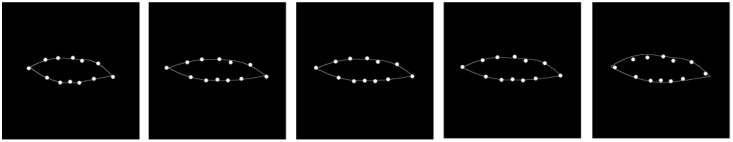
Example of model fits at five time points during specific trajectory for a single subject via mPCA for the frontal/coronal plane. (Increasing time is shown from left to right.) Points for the raw data are shown by the filled circles and the model fits are given by the curved lines.

Results for the mean smile trajectory and also the first mode of variation at level 1 via mPCA are shown in Figure 10. Note that these results are shown by full lines in Figure 10 (and similarly in Figure 11 and Figure 12), which are in fact two cubic spline fits for the upper and lower boundaries of the mouth separately with respect to the 12 points from the mPCA model in the frontal/coronal and horizontal planes, respectively. Again, this might lead to some additional smoothing, although this is carried out simply to make it easier to visualize and interpret model results. (Again, we believe that this is reasonable in these initial calculations.) The results shown in Figure 10 with respect to time are subtle to interpret. However, it is possible to see from a “movie” of shape changes with respect to time for the mean trajectory that the corners of the mouth are pulled outwards and backwards slightly as time evolves, before returning somewhat close to the original shape at the end of the smile. The first mode of variation at level 1 (between sexes) is definitely subtle; this mode is quite weak in magnitude and appears to govern the width of the lips and relative positions of upper and lower lips. A “movie” of shape changes with respect to time of the trajectory for the mean plus or minus this mode indicates again that the corners of the mouth are pulled outwards and backwards slightly as time evolves, before returning somewhat close to the original shape at the end of the smile (with some increased noise right at the end).

Results for the mean smile trajectory and the first mode of variation at level 2 are shown in Figure 11. The results are much easier to interpret than those in Figure 10. We see that this mode is clearly governing whether the natural “resting” mouth shape is either upturned or downturned. A “movie” of shape changes with respect to time of the trajectory for the mean plus or minus this mode indicates again that the corners of the mouth are pulled outwards and backwards slightly as time evolves, before again returning somewhat close to the original shape at the end of the smile (again with some increased noise right at the end). Note that this pattern with time is superimposed on upturned (mean minus this mode) or downturned (mean plus this mode) mouth shape appropriately for the entire trajectory, as illustrated in Figure 11.

Results in the frontal/coronal plane of the model fit to the twelve points for a specific person’s trajectory are shown in Figure 12. We see that excellent fits are obtained at all time points shown. Again, the time evolution is somewhat subtle for this case, although a “movie” of points and model fits indicates that the width of the mouth increases very slightly with time and then starts to reduce at the end. These results for the Smile dataset have been another excellent test of the mPCA method for “real” data.

## 4. Discussion

Multilevel PCA (mPCA) was used here to model dynamical changes in biological shapes. Results for simulated data for a single variable with two groups and for “blink” data with 32 variables (16 2D points) and two groups were shown to be modeled correctly, i.e., trajectories appeared correct, and the magnitude of eigenvalues made sense given the data generation model. The two dynamic trajectories of “surprise” and “blinking” were modeled adequately using the same multilevel model and strong differences between these trajectories were evident in both single-level PCA and also at level 1 of the mPCA model, as expected (and required). This is an encouraging first step.

However, this is exactly what one would expect for such simple simulated datasets. It is therefore important to test the method also for “real” data. This was provided here via the “smile” dataset of Refs. [11,12], consisting of 12 points placed on the outer boundary of the lips during entire smile trajectories for 29 females and 31 males. Again, dynamic smiles appeared to be modeled correctly when compared to the original data. Interestingly, differences between males and females appeared small in terms of magnitude of variation, as were differences between groups for standardized component scores. Indeed, there is no reason to suppose that males and females smile in fundamentally different ways (i.e., a smile is just a smile) and so this is an excellent test of “no effect” in terms of differences between groups. Broadly, we take all of these results as an excellent test of the method; both single-level PCA and mPCA can be applied to model shapes dynamically.

For between-group (bgPCA) [4,13,24], PCA is carried out separately with respect to (between-group) covariance matrices based on “group means” and (within group) covariance matrices based on individual shapes around these means; note that two-level mPCA is equivalent to bgPCA. A limitation of mPCA (as in bgPCA/mPCA) is that small numbers of groups can limit the number of non-zero eigenvalues (the rank of covariance matrices is reduced) at higher levels of the model. Another well-known “pathology” of bgPCA and multilevel PCA is that small sample sizes can lead to spurious differences between groups [4,13,24] because small differences between groups in terms of the positions of all points can become concentrated by reduced dimensionality. Although this can occur for standard PCA, this effect is more pronounced for bgPCA and mPCA because both are essentially a form of “guided” dimensionality reduction where differences between groups are concentrated at one specific level of the model. Full and detailed expositions of the “pathologies” of bgPCA are presented in Refs. [4,24], and the interested reader is referred to these articles for more information. However, various techniques [24,25,26] have been proposed to address such effects, including cross validation [25]. Monte Carlo simulations in Ref. [13] suggest that the number of subjects per group should be at least equal to the number of parameters (here, the number of points is multiplied by spatial dimensionality).

Clearly, the approach considered here relies on spline fits with respect to time to produce trajectories for the subsequent estimation of shapes at specific time points (here, time points that are sampled regularly over some overall period). Indeed, many of the practical aspects of implanting PCA appear to depend on the choice of method of curve fitting/smoothing procedures [14]. Here, we wished only to carry out a proof-of-principle of method and so we used a simple (cubic) spline fit (in MATLAB) to the data for each point component separately. This appeared to work well for both the simulated and real data, established by visual inspection of model fits to the (test) data.

There are many advantages to using a multilevel approach, especially for trajectories of dynamical shapes where paths for different groups (e.g., facial expressions for subjects with and without facial paralysis and/or for different types of expression) are likely to be radically different. In these cases, a multilevel approach should provide a more efficient and effective model because average trajectories for each group are found explicitly. Furthermore, mPCA allows us to quantify and explore differences between groups. For example, it was shown in this article that differences in shape between phases of a smile are likely to be large, whereas overall differences in how people smile dynamically is likely to be small between males and females (as expected). Finally, we believe that models that take into account differences between groups explicitly (e.g., images or shapes from different types of scanners), as well as differences within groups, might generalize more effectively than those that do not.

Note also that we previously either considered time as an implicit variable only (e.g., during the act of smiling [11,12]) or we used partial-least squares approaches to model shape changes as a (linear or quadratic) function of time, which should work well for cases where these changes are more gradual (e.g., facial shape changes with age in adolescents [8]). Here, splines fits were used in order to provide dynamical shapes sampled at regular time points, which should even work well when time-dependent changes are very strong (e.g., during facial expressions). Such spline fits should, in principle, reduce random errors by smoothing. In some cases, data frames might be missing, or data might be captured at irregular time points; spline fit data might be sampled at regular specific time points by interpolation. Thus, the method presented here is a step forward in treating dynamical changes in shapes and will hopefully lead to a “full” functional treatment of dynamical shapes [27] in due course. Future work will also concentrate on validating any such new methods and studying other types of dynamical objects. If we use multilevel approaches for multivariate data, similar issues of “pathologies” inherent in mPCA might occur and we will explore this also. Finally, ideas of clustering and hierarchies in the image or subject set will be explored also in the context of Deep Learning.

## Figures and Tables

**Figure A1:**
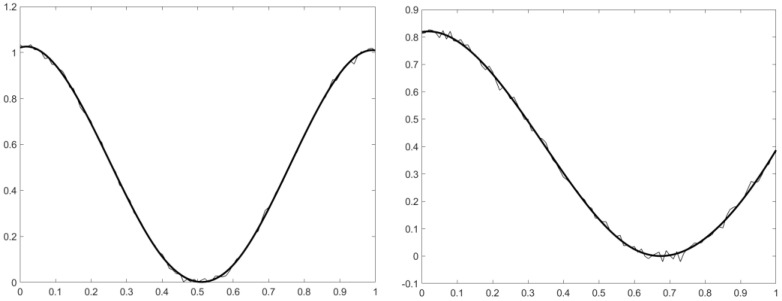
Examples cubic spline fits (thicker lines) to the MC data (thinner lines) for the Sine Wave dataset: (**left**) single trajectory for group 1; (**right**) single trajectory for group 2. Good model fits from the cubic splines are found in all cases.

**Figure A2:**
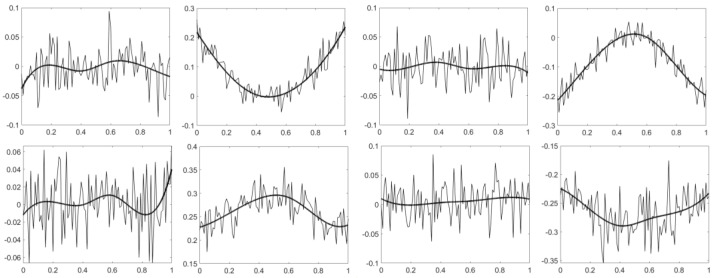
Examples cubic spline fits (thicker lines) to the MC data (thinner lines) for the Blink dataset. From to left to right: first column, y-positions of the 1st point (left corner of the eye); second column, 5th point (middle upper boundary); third column, 9th point (right corner of the eye); and fourth column, 13th point (middle lower boundary). Upper row is for the “blink” group, shown by y going to zero for points 5 and 13 at time 0.5 (arbitrary units of time); lower row is for the eyes widening/“surprise” group, shown by y increasing in magnitude for points 5 and 13 at time 0.5.

**Figure A3:**
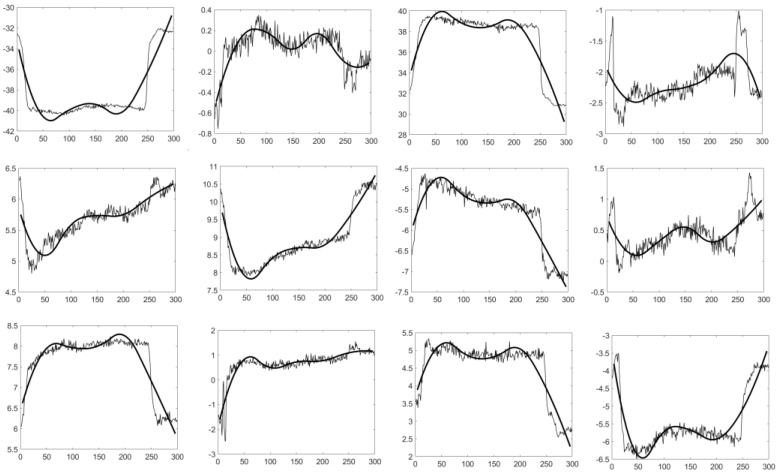
Examples cubic spline fits (thicker lines) to the real data (thinner lines) for the Smile dataset. From to left to right: first column, y-positions of the 1st point (left corner of the mouth); second column, 4th point (middle upper boundary); third column, 7th point (right corner of the mouth); and fourth column, 10th point (middle lower boundary). Upper row includes the x-components, middle row includes the y-components, and bottom row includes the y-components. The smile is evidenced (e.g.,) by the corners of the mouth for the 1st and 7th points being drawn outwards (x-components increase in magnitude) and backwards (z-components increase in magnitude).

**Figure A4:**
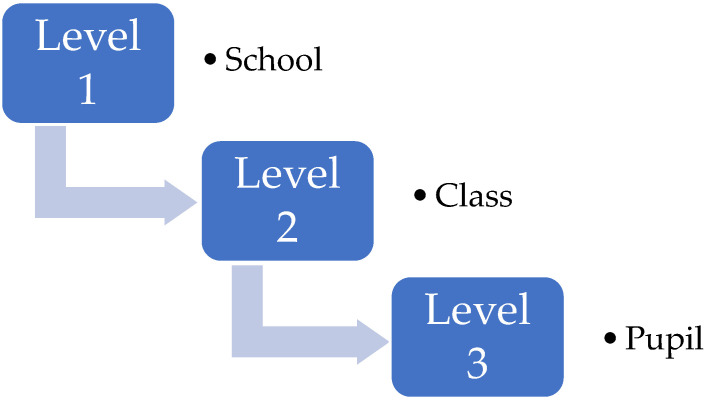
Example of a nested model—here, for (say) marks in a test for pupils in a school. Individual pupils are at the lowest level (level 3), where each pupil belongs to just one class in level 2. Furthermore, each class belongs to just one school. Variation can occur at all levels of the models: between pupils in each class; between classes in each school; and between schools.

## Data Availability

Not applicable.

## Appendix A

Here, we carry out simple cubic spline fits of X with respect to time for each subject separately at T regular time intervals in these initial calculations. Spline fits using the “spasp2” command in MATLAB were found to provide reliable trajectories (i.e., cubic splines fitted to data via a least-squares approach). Note that five “knots” gave reliable results without overfitting for these curves (assessed visually—this is adequate for these initial proof-of-principle calculations). Results for the “Sine Wave” dataset are shown in Figure A1 for two cases from the two groups. We see that these curves fit the data well (albeit for this very simple case.)

With respect to these multivariate data, we again carry out carry out simple cubic spline fits of X with respect to time for each subject separately at T regular time intervals. These initial calculations may then be sampled regularly at T time points for each spline fit for each component. The simplest method is now to let $X^{i}$ be an element of a concatenated vector of each of the Dim×M components of the shape of dimension Dim (Dim=2,3 here for 2D and 3D point data, respectively) and at all T time points for each subject i.

Results for the “Blink” dataset are shown in Figure A2 for components for two cases from each group. Similarly, results for the “Smile” dataset are shown in Figure A3. We see that these spline curves fit the data well in Figure A2 for the Blink data, even in the presence of considerable noise. The difference between the two modes (blinking and surprise) is evidenced by the trajectories of the middle points of the eye, whereas the corners of the eye change little (as specified for this MC data). Curves from spline fits match the raw data reasonably well for the Smile data in Figure A3, although the final neutral or “rest” phase is not captured particularly well for this particular subject in Figure A3. However, the broad deformations due to a smile are clearly being captured (e.g., evidenced by the corners of the mouth being drawn outwards and backwards); the purpose here is not to provide a perfect simulation in this case, but rather to prove the principle that these methods can be used. Clearly, optimized procedures for smoothing and interpolation could be used, and this will be an important part of future work.

## Appendix B

Again, we assume initially that the outcome is scalar and that trajectory data have been sampled regularly at T time points. For 3-level mPCA, elements of the covariance matrix at level 3 for each group j are given by

(A1) $$K_{s,t}^{3,j}=\frac{1}{n_{j}-1}\sum_{i}^{n_{j}}\left(X_{s}^{i,j}-\eta_{t}^{3,j}\right)\left(X_{t}^{i,j}-\eta_{t}^{3,j}\right).$$

$\eta^{3,j}$ is the mean for each group j at level 3, given by

(A2) $$\eta^{3,j}=\frac{1}{n_{j}}\sum_{i}^{n_{j}}X^{i,j}.$$

The “common” covariance matrix at level 3 is defined by

(A3) $$K^{3,j}=\frac{1}{p_{3}}\sum_{j}^{p_{3}}K^{3,j}.$$

Note that there are $p_{3}$ groups in total at level 3. The analysis has, thus far, followed that of the 2-level model. For nested mPCA, we now notice that means themselves may belong to specific groups in the level above them (e.g., classes in specific schools as shown in Figure A4). The means at level 2 are

(A4) $$\eta^{2,k}=\frac{1}{n_{k}}\sum_{j}^{n_{k}}\eta^{3,j}.$$

$n_{k}$ is the number of groups at level 3 that belong to the specific group k in level 2. Elements of the the covariance matrix at level 2 for each group k are given by

(A5) $$K_{s,t}^{2,k}=\frac{1}{m_{k}-1}\sum_{ij}^{m_{k}}\left(\eta_{s}^{3,j}-\eta_{s}^{2,k}\right)\left(\eta_{t}^{3,j}-\eta_{t}^{2,k}\right).$$

The “common” covariance matrix at level 2 is defined by

(A6) $$K^{2}=\frac{1}{p_{2}}\sum_{k}^{p_{2}}K^{2,k}.$$

Note that there are $p_{2}$ groups at level 2. The “grand mean” at level 1 is now given by

(A7) $$\eta^{1}=\frac{1}{p_{2}}\sum_{k}^{p_{2}}\eta^{2,k}$$

Elements of the covariance matrix at level 1 are written, finally, as

(A8) $$K_{s,t}^{1}=\frac{1}{p_{2}-1}\sum_{k}^{p_{2}}\left(\eta_{s}^{2,k}-\eta_{s}^{1}\right)\left(\eta_{t}^{2,k}-\eta_{t}^{1}\right).$$

$\theta_{l}^{1}$, $\theta_{l}^{2}$, and $\theta_{l}^{3}$ are non-negative eigenvalues at levels 1, 2 and 3, and $\psi_{l,t}^{1}$, $\psi_{l,t}^{2}$, and $\psi_{l,t}^{3}$ are the associated eigenfunctions at these levels. Again, the number of non-zero eigenvalues at levels 1 and 2 will be limited to the finite numbers of groups at these levels. The expansion of any (new) dynamic shape $X_{t}^{new}\;$ is given by

(A9) $$X^{new}=\eta^{1}+\sum_{l}^{m_{1}}a_{l}^{1}\psi_{l}^{1}+\sum_{l}^{m_{2}}a_{l}^{2}\psi_{l}^{2}+\sum_{l}^{m_{3}}a_{l}^{3}\psi_{l}^{3}.$$

Again, $a_{l}^{1}$, $a_{l}^{2}$, and $a_{l}^{3}$ are scalar coefficients and $m_{1}$, $m_{2}$, and $m_{3}$ are set to be finite numbers. The coefficients $\left\{a_{l}^{1}\right\}$, $\left\{a_{l}^{2}\right\}$, and $\left\{a_{l}^{3}\right\}$ may again be obtained by using a global optimization procedure in MATLAB. The extension to four or more levels for a fully nested model (reflecting the data) follows the pattern as that established above. Similarly, multivariate data can also be tackled readily by concatenating shape vectors at all time points. Non-nested cases (where there is no natural “nested” order to the data) may be carried out for mPCA as described in an Appendix in Ref. [8]. An example of non-nested cases might be marks in a test for groupings by both school and sex; there is no natural order to either sex or school, though one might posit that clustering might be possible via both variables.
